# Social support, empathy and compassion fatigue among clinical nurses: structural equation modeling

**DOI:** 10.1186/s12912-023-01565-6

**Published:** 2023-11-13

**Authors:** Jie Zhang, Xiao Wang, Ouying Chen, Juan Li, Yifei Li, Yiping Chen, Yaoyue Luo, Jingping Zhang

**Affiliations:** 1 Hunan University of Chinese Medicine, 300 Xueshi Road, Changsha, Hunan 410208 China; 2 The Third Xiangya Hospital, Central South University, Changsha, Hunan 410013 China; 3 Nursing Psychology Research Center of XiangYa School of Nursing, Central South University, 172 Tongzi Po Road, Changsha, Hunan 410013 China; 4 Southern Medical University Shenzhen Hospital, Shenzhen, Guangdong 510086 China

**Keywords:** Compassion fatigue, Empathy, Social support, Mediating effect, Structural equation model

## Abstract

**Background:**

Clinical nurses are at high risk for compassion fatigue. Empathy is a prerequisite for compassion fatigue, and social support is an important variable in the process of reducing individual stress. However, the role of social support in the relationship between empathy and compassion fatigue remains unclear. This study explored whether social support mediates the relationship between empathy and compassion fatigue among clinical nurses.

**Methods:**

A total of 992 clinical nurses were recruited through convenience sampling for a cross-sectional study in Central China. They completed the General Information Questionnaire, Perceived Social Support Scale, Professional Quality of Life Scale, and Jefferson Scale of Empathy. SPSS was used to conduct descriptive statistical analyses. Pearson’s or Spearman’s correlation analyses and AMOS were employed to build a structural equation model (SEM) to verify the mediating effect of social support on the relationship between empathy and compassion fatigue.

**Results:**

The results indicated that the standardized direct effect of empathy on compassion fatigue was 0.127, and the standardized indirect effect of empathy on compassion fatigue through social support was 0.136. The mediation effect ratio between empathy and compassion fatigue was 51.7%.

**Conclusions:**

Our findings show that social support mediates the relationship between empathy and compassion fatigue among clinical nurses. This finding suggests that increasing nurses’ social support can decrease the prevalence of compassion fatigue. Nursing managers should provide training related to flexibly adjusting empathy and educating nurses to establish effective social networks with family, friends, and colleagues to prevent compassion fatigue.

**Supplementary Information:**

The online version contains supplementary material available at 10.1186/s12912-023-01565-6.

## Background

As direct providers of medical services, nurses play a key role in promoting patient health and alleviating nurse-patient conflicts. Nurses are more susceptible to compassion fatigue than other medical staff members [1]. Compassion fatigue is the process in which the caregiver endures the recipient’s pain with compassion, which reduces the recipient’s energy or interest in themselves [2], and Figley deemed that it is the “cost of caring” [3]. Several studies have shown that the incidence of compassion fatigue in clinical nurses ranges from 7.3–44.8%, which is very high [4], especially for nurses from psychiatric and oncology departments who suffer from severe compassion fatigue [5, 6]. Growing evidence suggests that compassion fatigue can influence nurses’ physical, social, emotional, spiritual, and cognitive aspects that endanger their viability [7]. Compassion fatigue is associated with a high incidence of anxiety and depression, increased clinical error rates, decreased performance, nursing quality, and job satisfaction [8, 9]. Therefore, compassion fatigue in nurses has received considerable attention. Consequently, managing compassion fatigue among clinical nurses is of special significance in maintaining their mental health.

According to the Compassion Stress and Fatigue Model [3] proposed by Figley, empathy is a keystone both to help others and to be vulnerable to the costs of caring, and empathy is a prerequisite for compassion fatigue. Empathy is the activity of understanding the situations, thoughts and feelings of another person from another person’s perspective, not from one’s own perspective [10] [11]. For nurses, empathy is the process by which they can put themselves in the patients’ shoes, perceive their emotions, comprehend their situation, and communicate these insights and understandings to patients [12]. Several studies explored the relationship between empathy and compassion fatigue. They verified that empathy is one of the basic conditions for compassion fatigue [13] and suggested that nurses’ empathy ability is significantly positively correlated with compassion fatigue; nurses with high levels of empathy ability are more likely to develop compassion fatigue [14]. Additionally, some studies have found that empathy, especially perspective taking, is a predictor of compassion fatigue in emergency nurses [15]. Empathy can affect compassion fatigue, but whether there is an indirect influence path remains unknown. Therefore, it is important to explore the direct and indirect factors affecting compassion fatigue in nurses.

According to the stress-coping model, as an important external resource when an individual is stressed, social support affects health outcomes [16]. Social support contains various forms of free social media assistance, including emotional and physical support, which can be formal or informal [17]. This can help people improve their problem-solving skills, promote adaptation to pressure, and reduce the influence of pressure on their physical and mental health. Studies have explored the correlation between social support and compassion fatigue. For example, Saeed surveyed 173 Iranian nurses and found that social support (significant others, friends, and family) was negatively associated with compassion fatigue, and increased social support contributed to worse compassion fatigue [18]. It was also reported that higher family support fostered more compassion satisfaction but less compassion fatigue among nursing students [19]. Conversely, several studies have explored the relationship between empathy and social support. Park evaluated the relationship between social support and empathy in medical students. Results showed that empathy and social support were positively correlated [20]. Research has also indicated that a potential chain reaction of social support and empathy in online mental health communities was produced, and users who received more support subsequently expressed a higher level of empathy for others in the future [21]. In summary, there is a direct or indirect relationship between empathy, social support, and compassion fatigue. Clarifying this relationship is of great significance in proposing new strategies to improve compassion fatigue among nurses.

Additionally, the psychological stress theory and the Compassion Stress and Fatigue Model guided this research. According to the psychological stress theory [22], social support can be used as an important mediating variable in the process of reducing individual stress and can affect the outcome of stressful events. Empathy is a prerequisite for compassion fatigue according to the Compassion Stress and Fatigue Model. Clinical nurses have long struggled with chronic work-related stress because they empathize with patients’ painful and traumatic experiences without getting adequate rest, which can be stressful situations. Social support may play a mediating role in this process, so compassion fatigue is the result of long-term stress in nurses’ work [4]. Based on these theories, we hypothesized that social support may play a mediating role between empathy and compassion fatigue.

The objectives of this study were to explore the levels of compassion fatigue, empathy, and social support in clinical nurses and test the role of social support in the relationship between empathy and compassion fatigue.

## Methods

### Design and setting

This was a cross-sectional descriptive study. This study was conducted in accordance with the STROBE Statement; that is, guidelines for reporting observational studies [23].

### Participants

Convenience sampling was used to recruit nurses from three tertiary hospitals in China. The inclusion criteria are as follows: (1) the hospital is a general hospital, (2) nurse registration and on-the-job, (3) the nurse is now engaged in clinical work, and (4) the nurse is willing to participate in the study. Interns, nurses trained in other hospitals or participating in other relevant studies are not included in the scope of this study.

It is generally recommended that the average value of structural equation model (SEM) analysis samples be 200 [24]. The prior sample size calculation of SEM is applied, which is a popular and general SEM sample size calculation calculator (https://www.danielsoper.com/statcalc/calculator.aspx?id=89). The minimum number of samples had a medium effect (0.3), including three potential variables and eight observed variables, with power of 0.95 and α.of. 05. Based on the calculations, it was 184. Considering a dropout rate of 10%, we selected 203 participants as the minimum sample size. This implies that a minimum sample size must be met in this study.

### Instruments

#### General information questionnaire

A general questionnaire was prepared to collect demographic characteristics of the participants, such as gender, age, education level, length of service in nursing, marital status, labor department, type of employment, professional title, monthly income, shift work, frequency of exercise, presence of children, and physical conditions.

#### Jefferson scale of empathy

This instrument was developed to assess the empathy status of empaths [25]. The Chinese version was translated [26]. It covers 20 items in three areas (compassionate care, perspective-taking, and standing in patients’ “shoes”). Each item consists of a seven-point scale (1= “absolutely disagree,” 7= “absolutely agree”), higher scores indicate greater empathy. On the original scale, Cronbach’s a was 0.80 [26]. In this study, Cronbach’s a was 0.762.

#### Perceived social support scale

This instrument was designed by Zimet [27]. This scale comprises three dimensions: Support from friends, support from family, and support from others. It is measured using a 7-point scores (7= “extreme consent” to 1= “extreme disgust”). The higher the overall score, the higher is the level of social support. This scale has good internal consistency and high reliability. Cronbach’s alpha for the scale in this study was 0. 957.

#### Professional quality of life scale (Chinese version; ProQOL-CN)

The instrument was designed by Stamm [28] and aimed to evaluate compassion fatigue. It was translated into Chinese by Zheng [29]. The scale contains 30 items and three subscales: compassion satisfaction, secondary traumatic stress, and burnout. The burnout and secondary traumatic stress subscales measure compassion fatigue [28]. The scale was measured by five-point Likert score (5 = “very often” to 1 = “never”) [30]. The higher the score, the higher the degree of compassion satisfaction and the higher the risk of secondary traumatic stress and burnout. The scores on each scale were lower than 22, indicating low levels of compassion satisfaction, burnout, and secondary traumatic stress; 23–41 suggests a medium level; and ≥ 42 indicates a high level [28]. This scale is widely used, with Cronbach’s alpha ranging from 0.76 to 0.80 [31], demonstrating acceptable internal reliability. In this study, the Cronbach’s alpha for the scale was 0.722.

### Data collection

Data were collected from October 3 to December 15, 2019. One researcher and two research assistants were responsible for the data collection. First, the researchers informed the directors and head nurses of the purpose of the study from each hospital and obtained their permission to recruit nurses. According to the standards, interested nurses could participate in the study by contacting researchers and research assistants in hospitals. Researchers sent links related to the electronic research questionnaire (Wenjuanxing, China’s online packaging platform) to a hospital research assistant. Wenjuanxing is a professional online questionnaire survey platform that can be used to design online questionnaires. After the completion of the questionnaire, a link was generated. Participants could fill in the data online by clicking on a link. Researchers could download data online through the platform for data analysis after the questionnaires were submitted. It should be noted that Wenjuanxing is a relatively safe platform with no risk of data loss or leak to third parties.

### Ethical considerations

Before completing the questionnaire, all eligible participants signed an electronic informed consent form. Participants took part voluntarily and anonymously. All the participants had the right to withdraw from the study at any time. This study was approved by the university’s institutional review board (IRB) before data collection (No: E202027).

### Data analysis

IBM SPSS Statistics (version 20.0; IBM, Chicago, IL, USA) and AMOS (version 20.0; IBM, Chicago, Illinois, USA) were used to conduct the statistical analyses. Descriptive data were used to analyze the demographic information and correlations between the two variables. The hypothesized model consisted of three latent variables (empathy, compassion fatigue, and social support) and eight observed variables (perspective-taking, standing in patients’ “shoes,” compassionate care, support from friends, support from family, support from others, burnout, and secondary traumatic stress). The comparative fit index (CFI), incremental fit index (IFI), normed fit index (NFI), goodness-of-fit index (GFI), and the root mean square error of approximation (RMSEA) were used as model fit indicators to verify the SEM. Values of NFI, CFI, GFI, and IFI > 0.90 are considered to reflect a good model fit. RMSEA values < 0.05 mean good fit, and values of 0.08 mean reasonable error and an acceptable fit [32].

## Results

### Sample profile

A total of 992 nurses participated in the study. However, only 978 nurses were included in the analysis (valid response rate of 98.6%), because 14 nurses declined to complete the questionnaires. Most nurses were women (93.9%), 64.8% of the nurses ranged from 26 to 35 years, and most participants had a bachelor’s degree. Other general information on the participants is presented in Table 1.

**Table 1:** Socio-demographic characteristics of participants (N = 978)

Variables	Category	N	%
**Age(years)**			
	20–25	215	22
	26–35	634	64.8
	≥ 36	129	13.2
**Gender**			
	Female	918	93.9
	Male	60	6.1
**Education level**			
	Secondary vocational school diploma	3	0.3
	Associate degree	119	12.2
	Bachelor degree	784	80.2
	Master degree or above	72	7.4
**Marital status**			
	Married	599	61.2
	Single	366	37.4
	Divorced or separated	13	1.3
**Department**			
	Medical	271	27.7
	Surgical	237	24.2
	Obstetrics and Gynecology	63	6.4
	Pediatrics	29	3.0
	Emergency departments	23	2.4
	ICU	71	7.3
	Operating room	98	10.0
	Outpatient services	54	5.5
	Psychiatry	10	1.0
	Oncology	18	1.8
	Others	104	10.6
**Years of nursing** **experience**			
	< 2 years	141	14.4
	2-5years	229	23.4
	6–10 years	346	35.4
	11–20 years	202	20.7
	21–30 years	46	4.7
	≥ 31 years	14	1.4
**Professional title**			
	Junior RN	202	20.7
	Senior RN	512	52.4
	Nurse in charge	237	24.2
	Associate professor or professor nurses	27	2.8
**Employment type**			
	Formal employed nurse	185	18.9
	Personal agent nurse	538	55.0
	Contract employed nurse	255	26.1
**Income per month**			
	< 3,000 yuan (US, $500)	55	5.6
	3,001–5,000 yuan (US, $500–$830)	120	12.3
	5,001–7,000 yuan (US, $830–$1,160)	254	26.0
	7,001–9,000 yuan (US, $1,160–$1,500)	329	33.6
	> 9,001 yuan (US, $1,500)	220	22.5
**Shift work**			
	Yes	658	67.3
	No	320	32.7
**Have any children**			
	Yes	515	52.7
	No	463	47.3
**Frequency of exercise**			
	Never	226	23.1
	Sometimes	668	68.3
	Always	84	8.6
**Physical conditions**			
	Good	370	37.8
	General	497	50.8
	Bad	111	11.3

### Empathy, compassion fatigue, social support, and their associations

The average total scores for empathy and social support were 79.31 (SD = 4.51) and 59.30 (SD = 12.61), respectively. The average values for compassion satisfaction, secondary traumatic stress, and burnout were 31.97 (SD = 7.20), 27.15 (SD = 5.54), and 27.49 (SD = 5.31), respectively. Detailed illustrative results are presented in Table 2.

**Table 2:** Mean and standard deviations of variables (N = 978)

Variables	Mean	SD
**Compassion satisfactory**	31.97	7.200
**Compassion fatigue**	54.63	9.242
Burnout	27.49	5.314
Second traumatic stress	27.15	5.545
**Empathy**	79.31	4.506
Perspective-taking	38.30	2.956
Compassionate care	29.88	3.194
Standing in the patient’s shoes	11.13	2.185
**Social support**	59.30	12.605
Family support	20.03	4.854
Friend’s support	19.84	4.354
Other support	19.44	4.509

Regarding the association among the variables, empathy (r = 0.132, p < 0.05) was significantly positively correlated with compassion fatigue, whereas social support had a significantly negative association with compassion fatigue (r = -0.323, p < 0.05) and empathy (r = -0.146, p < 0.05). Detailed information is provided in Table 3.

**Table 3:** Pearson’s correlations (p-values) between variables among nurses (N = 978)

	Compassion fatigue (r, p)	compassion satisfaction (r, p)	Empathy (r, p)	Social support (r, p)
**Compassion fatigue**	1			
**compassion satisfaction**	-0.392(p < 0.001)*	1		
**Empathy**	0.132(p < 0.001)*	-0.083(p < 0.001)*	1	
**Social support**	-0.323(p < 0.001)*	0.477(p < 0.001)*	-0.146(p < 0.001)*	1

**Note**: * p < 0.05

### Structural equation model of the three variables

According to Wen’s rules of mediate effect [33], we first use SPSS to test the mediating role of social support in the interpersonal relationships between empathy and compassion fatigue. Using compassion fatigue as the dependent variable, empathy as the independent variable, and social support as the mediating variable, three regression analyses are performed. Step 1: Empathy can significantly predict compassion fatigue (β = 0.132, p < 0.001); Step 2: Empathy significantly affects social support (β=-0.146, p < 0.001); Step 3: After including social support variables, empathy still has a significant impact on compassion fatigue (β = 0.087, p < 0.05), and when social support is included in the regression equation, the regression coefficient of empathy decreases, indicating that social support has a partial mediating effect on the relationship between empathy and compassion fatigue.

**Fig. 1:**
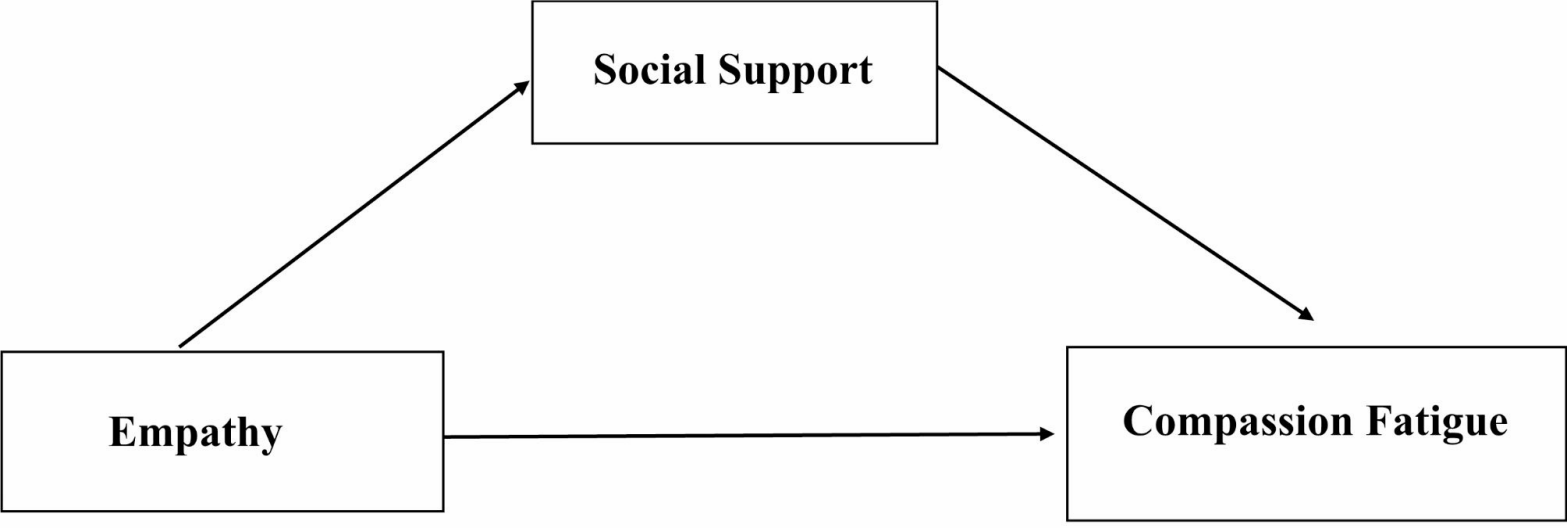
The hypothesis model

**Fig. 2:**
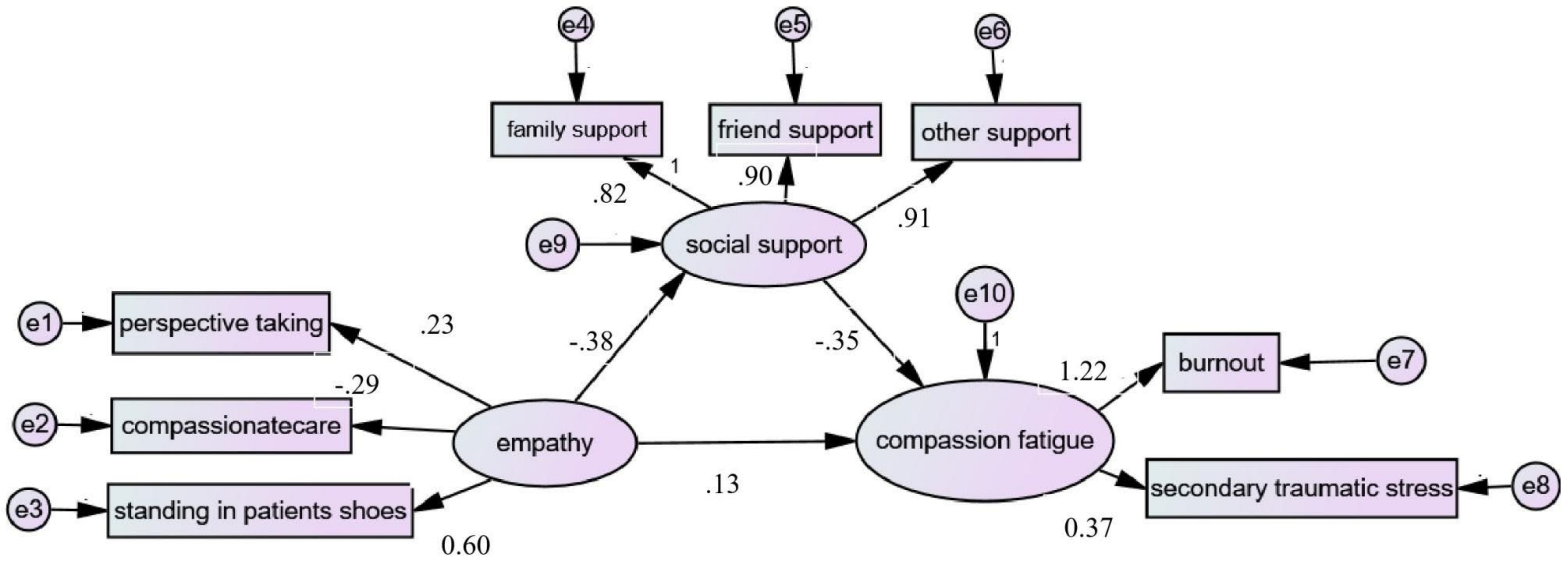
The validated model

To present the mediating role of social support more intuitively, we adopt AMOS to verify the hypothesis model (Fig. 1). The CFI, IFI, NFI, GFI, and RMSEA values suggested that this model fit the data well (Fig. 2). The detailed fitted indices are presented in Table 4. As the model shows, the standardized direct effect of empathy on compassion fatigue was 0.127, and the standardized indirect effect of empathy on compassion fatigue through social support was 0.136. This means that social support has a partial mediating effect on the relationship between empathy and compassion fatigue, with a mediation effect ratio of 51.7%. Table 5 shows the overall, standardized direct and indirect effects of each variable, and Table 6 shows the maximum likelihood estimates of the model.

**Table 4:** Comparison of model fit for the modified model to the hypothetical model

Model	χ2 (P)	df	χ2/ df	GFI	NFI	IFI	CFI	RMSEA
Reference	> 0.05		< 5	0.9-1	0.9-1	0.9-1	0.9-1	< 0.08
Fitted model	71.048 (0.00)	17	4.179	0.982	0.973	0.980	0.979	0.057

Abbreviations: GFI, goodness-of-fit index; NFI, normed fit index; IFI, Incremental Fit Index; CFI, comparative of fit index; df, degree of freedom; RMSEA, root mean square error of approximation;

**Table 5:** Standardized direct, indirect, and total effects for the modified model

Path	Standardized Direct Effects	Standardized Indirect Effects	Standardized Total Effects
Empathy → Compassion Fatigue	0.127	0.136	0.263
Social support → Compassion Fatigue	-0.354	/	-0.354
Empathy → Social Support	-0.384	/	-0.384

**Table 6:** Maximum likelihood estimates of the fitted model

Pathway	Non-standardized Coefficients	Standardized Coefficients	Standard Errors	Critical Ratio	P
Social Support ← Empathy	-1.169	-0.384	0.279	-4.195	< 0.001*
Compassion Fatigue ← Empathy	0.634	0.127	0.245	2.584	0.010*
Compassion Fatigue ← Social Support	-0.580	-0.354	0.051	-11.313	< 0.001*

**Note**: * p < 0.05.

## Discussion

Reducing compassion fatigue is of great significance for maintaining the physical and mental health of clinical nurses, improving the quality of patient care, and improving the nurse-patient relationship. This study explored the effects of social support on the relationship between empathy and compassion fatigue (secondary traumatic stress and burnout) and examined the relationships among the three variables in nurses. Our results are intended to deepen the understanding of providing more social support to nurses to relieve their compassion fatigue.

### Status of compassion fatigue and empathy among Chinese nurses

Secondary traumatic stress together with burnout increases the risk of compassion fatigue [28]. In this study, clinical nurses had an average level of burnout and secondary traumatic stress, which were higher than the levels of nurses from Iran [31] and American emergency departments [34]. Yu surveyed 186 nurses from an emergency department in China and found that nurses in the emergency department experienced lower levels of secondary traumatic stress and burnout compared to the results of this study [15]. In contrast to previous research on clinical nurses in different nursing departments [35], our results show similar levels of burnout but higher secondary traumatic stress. The reasons for these differences may be attributed to different national conditions, working environments, departments, workloads, and the severity of the patient’s condition. Therefore, it also reminds us that Chinese nurses are experiencing a high level of compassion fatigue. We should pay attention to this problem and take appropriate measures to actively help them cope. As a precondition for compassion fatigue, the empathy ability of nurses in this study was 79.31(SD = 14.6), which was relatively lower than that of nurses working in emergencies, critical care units, and psychiatric wards in Iran [36]. Previous research has indicated that the empathy ability of nurse practitioners in the medical field ranges from 104 to 140 points. Additionally, Hui [37] investigated 733 nurses and found that their level of empathy was 97.6 (SD = 14.6), which was higher than the results of this study. Possible reasons for these differences include regional differences, department differences, the tighter relationship between nurses and patients in recent years, and the increase in medical violence experienced by nurses. Our study also suggests that if nurses have a high level of empathy for patients, they are at greater risk of compassion fatigue. This also reminds us of the need for strengthening clinical nurses’ training in using empathy in a reasonable and flexible manner.

### Mediating role of social support between empathy and compassion fatigue

Previous studies have shown that empathy has wide-ranging benefits in nursing practice, including improving clinical outcomes and patient satisfaction, promoting relationships between nurses and patients, and enhancing the quality of nursing [38]. However, empathy requires imaginative experience of the patient’s situation and is, therefore, emotionally draining. Empathy is a prerequisite for compassion fatigue according to the Compassion Stress and Fatigue Model [3], and nurses are at a high risk of compassion fatigue when they face patients with severe physical and psychological distress or when those who are dying have strong demands for empathic care. Therefore, empathy is a double-edged sword. Several studies have indicated that empathy has a positive relationship with compassion fatigue, and that nurses with high levels of empathy are more likely to suffer from compassion fatigue [39],which is consistent with the results of this study. Therefore, nursing administrators should recognize the importance of empathy, develop empathy training programs to instruct nurses to use it wisely, properly view patients’ perspectives and feelings, and cultivate a more compassionate environment in which nurses can avoid the risk of compassion fatigue.

The results of this study showed that the higher the level of social support, the lower the level of compassion fatigue among nurses, which is consistent with the results of Ariapooran [18]. Previous research also found that social support showed a significant negative correlation with secondary traumatic stress (part of compassion fatigue) and influenced secondary traumatic stress [40]. Ren surveyed 335 frontline nurses during the COVID-19 epidemic and indicated that social support played a mediating role between psychological resilience and compassion fatigue, which showed a significant effect of social support on compassion fatigue [41]. Additionally, social support from family and friends significantly affects the physical and mental health of nurses [42]. In their daily work, nurses often deal with patients’ traumatic experiences and empathize with them. After a long period, coupled with insufficient rest, they experience emotional exhaustion. If nurses can access different forms of support from family, friends, and colleagues, they can boost their confidence and courage to solve problems, prompting them to use positive response methods, such as talking to relatives or friends or engaging in different forms of relaxation activities with family and friends to relieve negative emotions and prevent compassion fatigue. Clinical nurses empathize with patients but do not adjust themselves in time, which can directly lead to compassion fatigue but can also indirectly weaken the risk of compassion fatigue through social support. Therefore, it is recommended that nursing managers build an effective social support network for nurses, guide them in finding social support around them when facing work pressure, and adopt active coping strategies to relieve compassion fatigue in clinical nurses.

### Limitations

This study had some limitations. First, this was a cross-sectional study, and a causal relationship between the variables could not be drawn. A follow-up study is recommended to verify the cause-effect relationships among these variables in nurses. Second, convenience sampling was adopted in this study. The samples were insufficiently representative, and the results may not be generalizable to populations in other geographic regions. Future studies should recruit nurses through random sampling to promote generalizability. Third, this study collected the data online. Owing to the difference between the Internet and mobile devices, some items of the questionnaire may have been incorrectly analyzed by participants, which may have led to deviations in the collected data. Future research should focus on screening and verifying online data. Finally, the relationship between empathy and compassion fatigue is complex and there may be other intermediary variables. Future research should explore other intermediary variables to provide empirical support for compassion fatigue interventions.

### Implications

The findings of this study provide new insights to assist in developing effective strategies to prevent compassion fatigue in clinical settings and maintain the mental health of nurses. We should formulate nursing policies, develop supportive working environments, and support networks for clinical nurses, and guide them in finding social support when facing work pressure to adopt active coping strategies. Training programs such as empathy skills training are recommended for nurses to flexibly use empathic capacity in nursing.

## Conclusions

The findings showed that clinical nurses experienced moderate levels of compassion fatigue (burnout and secondary traumatic stress) and a low level of empathy. Empathy was significantly positively correlated with compassion fatigue, whereas social support was significantly negatively associated with compassion fatigue and empathy. Social support may also partially explain the association between empathy and compassion fatigue. Hospital administrators, policymakers, and nurse leaders should be aware that both empathy and social support influence compassion fatigue.

## Electronic supplementary material

Below is the link to the electronic supplementary material.

Supplementary Material 1

## Data Availability

The datasets supporting the conclusions of this article are included within the article.
