# Correction: Social support, empathy and compassion fatigue among clinical nurses: structural equation modeling

**DOI:** 10.1186/s12912-024-02432-8

**Published:** 2024-10-14

**Authors:** Jie Zhang, Xiao Wang, Ouying Chen, Juan Li, Yifei Li, Yiping Chen, Yaoyue Luo, Jingping Zhang

**Affiliations:** 1 Hunan University of Chinese Medicine, 300 Xueshi Road, Changsha, Hunan 410208 China; 2 The Third Xiangya Hospital, Central South University, Changsha, Hunan 410013 China; 3 Nursing Psychology Research Center of XiangYa School of Nursing, Central South University, 172 Tongzi Po Road, Changsha, Hunan 410013 China; 4 Southern Medical University Shenzhen Hospital, Shenzhen, Guangdong 510086 China

**Correction: BMC Nursing (2023) 22:425**

10.1186/s12912-023-01565-6

The authors of the original article wish to acknowledge that Dr. Mohammadreza Hojat of Thomas Jefferson University was the creator of the Jefferson Scale of Empathy used in the original article.

